# Safety of Pegfilgrastim (Neulasta) in Patients with Sickle Cell Trait/Anemia

**DOI:** 10.1155/2013/146938

**Published:** 2013-12-11

**Authors:** Pashtoon Murtaza Kasi, Mrinal M. Patnaik, Prema P. Peethambaram

**Affiliations:** ^1^Hematology/Oncology, Mayo Clinic, Rochester, MN 55905, USA; ^2^College of Medicine, Mayo Clinic, Rochester, MN 55905, USA

## Abstract

Pegfilgrastim (Neulasta) is a recombinant filgrastim (human granulocyte colony-stimulating factor (G-CSF)) attached to a polyethylene glycol (PEG) molecule and is given as part of chemotherapy regimens that are associated with significant myelosuppression and risk for febrile neutropenia. Prescribing information available on manufacturer's website for the drug warns us about possible severe sickle cell crises related to the medication but does not report the actual incidence or the use in patients with sickle cell trait. Caution is advised when using it in patients with sickle cell disease. Here we present a case of a Caucasian female with known sickle cell trait (SCT) with no prior complications who developed a presumed sickle cell crisis after getting Neulasta, as a part of the chemotherapy regimen used to treat her breast cancer. Based on our literature review, this appears to be the first case report of a patient with SCT developing a sickle cell crisis with the pegylated form of recombinant filgrastim. Given the dearth of literature regarding the use of G-CSF and its related pegylated forms in patients with sickle cell anemia and sickle cell trait, a discussion of potential mechanisms and review of current literature and guidelines is also presented.

## 1. Background

Pegfilgrastim is a recombinant filgrastim (human granulocyte colony-stimulating factor (G-CSF)) attached to a polyethylene glycol (PEG) molecule and is given as part of chemotherapy regimens that are associated with significant myelosuppression and febrile neutropenia [1]. Prescribing information available on manufacturer's website for Pegfilgrastim warns us about possible severe sickle cell crises due to the medication but do not report the actual incidence or the use in patients with sickle cell trait [2]. Caution is advised when using it in patients with sickle cell disease [3].

The heterozygous form of sickle cell anemia, sickle cell trait (SCT), is present in up to 8% of African-Americans in the United States [4, 5]. Higher prevalence of up to 25% has been reported in some populations, but it is very rare to find sickle cell disease or trait in Caucasians [6, 7]. Although considered a benign disorder, it has been associated with numerous complications and adverse events, more so when exposed to conditions that would promote sickling, for example, periods of stress or high altitude [6, 8]. Case reports ranging from pain crisis to rhabdomyolysis, renal failure, and splenic/intestinal infarction have been reported [9, 10].

With respect to administration of colony stimulating factors in patients with sickle cell trait, as pointed out by Kang and colleagues, “the use of G-CSF may represent another form of stressor” [11]. Here we present a case of a patient with sickle cell trait with no prior complications who developed a sickle cell crisis after getting the pegylated form of filgrastim. Based on our literature review, this appears to be the first case report of a patient with sickle cell trait developing a sickle cell crisis with the pegylated form of recombinant filgrastim (human granulocyte colony-stimulating factor (G-CSF) Neulasta).

## 2. Case Presentation

A 54-year-old Caucasian woman with sickle cell trait and no other comorbidities presented to the medical oncology clinic for further management of her recently diagnosed breast cancer.

Her oncological history dates back to March 2013 when she initially underwent a left total mastectomy with sentinel lymph node biopsy for an infiltrating ductal carcinoma, grade 3 (of 3), measuring 1.4 × 1.2 × 0.9 cm in the upper outer quadrant of the left breast. There were two other separate foci of ductal carcinoma in situ, low nuclear grade, involving radial scars: one in the lower outer quadrant measuring 0.7 × 0.5 cm and one in the central aspect measuring 0.5 × 0.5 cm. There was no angiolymphatic invasion. All the margins were noted to be negative for tumor with the nearest tumor-free margin of 1.7 cm for the in-situ carcinoma and 1.8 cm for the invasive carcinoma. Three sentinel lymph nodes were noted to be negative for tumor. Estrogen Receptor and Progesterone Receptor status were checked on the specimen and were negative with less than 1% tumor nuclei staining. HER-2/neu overexpression was negative with a score of 1+. Ki-67 was 81.4%.

Given the profile, adjuvant Docetaxel (Taxotere) and Cyclophosphamide (Cytoxan) was started, April 18, 2013. The first cycle of chemotherapy was complicated by neutropenic fever requiring hospitalization; therefore, Pegfilgrastim (Neulasta) was added to her regimen. Cycles two and three were essentially uneventful. In June, after receiving her fourth and final cycle of Docetaxel and Cyclophosphamide followed by Neulasta she developed shortness of breath, severe substernal chest pain, and diffuse body aches. Due to persistent symptoms she was hospitalized. Cardiac enzymes, a transthoracic echocardiogram, and a CT scan of the chest PE protocol were normal. There was no evidence for infection. Her CBC revealed a hemoglobin of 11.2 g/dL on day of admission with a white count of 17.4 × 10^9^/L, with 16.9 × 10^9^/L neutrophils.

Given her history of sickle cell trait and her presentation mimicking a sickle cell crisis, she was given supportive therapy in the form of intravenous hydration and pain control to which she responded well [12, 13].

## 3. Discussion

There is a dearth of literature regarding the use of G-CSF and its related pegylated forms in patients with sickle cell anemia and sickle cell trait. Mechanisms postulated include the sudden elevation in granulocytes, neutrophil activation, and cytokine release along with possible increased leukocyte adhesion leading to a cascade of events causing vasoocclusion in patients with sickle cell anemia [14, 15]. Sickle cell related complications have been reported even when G-CSF was given intralesionally in patients with sickle cell related ulcers [16].

Table 1 summarizes similar cases of patients with sickle cell anemia and complications attributed to G-CSF administration [17]. Of note, in a prospective trial of 8 patients with sickle cell trait and matched controls, successful mobilization of peripheral blood stem cells was achieved with G-CSF administration without any major complications reported [11]. However, it is interesting to note that in the same study, patients with SCT had a higher cumulative symptom score (3.44 versus 1.25), which was statistically significant (*P* value = 0.014). Most common symptoms reported included nausea, headaches, myalgias, and fatigue. The average use of analgesics was also higher (13 tablets versus 6.25 tablets) in patients with SCT versus controls, respectively, suggesting a possible association between G-CSF use and sickle cell crises in SCT patients. Grigg and colleagues postulated that there may be a threshold HbS level beyond which it is likely that patients with sickle cell trait would have complications with G-CSF administration [15]. In the prospective trial by Kang and colleagues, the HbS levels in their patient population was noted to be 35–40%, whereas in the case of the sickle cell crisis reported by Griggs, the HbS level was 57% [11, 15]. The same association, however, was not noted by a study from Chicago on 5 patients with sickle cell disease undergoing peripheral blood stem cells mobilization [18].

In patients with sickle cell trait or for that matter sickle cell disease requiring growth factor support, close monitoring is required. This is even more important in the current era where colony stimulating factors are used for peripheral blood stem cells (PBSC) mobilization in hematopoietic stem cell transplant donors and in patients with sickle cell anemia requiring myelosuppressive chemotherapy for other neoplasms where growth factor support may be needed [11, 19].

Although there are no clear guidelines or a particular “threshold” for leucocytosis or HbS levels to induce crises, there is some suggestion that it is the best to avoid G-CSF in patients with HbS levels of more than 30% and to stop therapy if there is significant leukocytosis (>80 × 10^9^/L) [17, 18]. In patients developing significant leucocytosis and crises, interventions aiming at reducing the white blood cell counts may be worth considering [12]. Split dose schedule schedules and exchange transfusions to reduce the levels of HbS have also been shown to be effective in isolated cases [19–22]. It would also be advisable that if after consideration of risks and benefits, the use of filgrastim and pegfilgrastim is indicated, that it be done in centers equipped with dealing with patients with sickle cell anemia.

## 4. Conclusions

- Patients with sickle cell trait also are prone to developing sickle cell crises when exposed to stressors predisposing the cells to sickling.
- Although there are no clear guidelines, there is some suggestion to avoid G-CSF in patients with HbS levels of more than 30% and to stop therapy if there is significant leukocytosis (>80 × 10^9^/L).
- Split dose schedule schedules and exchange transfusions to reduce the levels of HbS have also been shown to be effective in isolated cases.
- It would be advisable that if after consideration of risks and benefits, the use of filgrastim and pegfilgrastim is indicated, that it be done in centers equipped with dealing with patients with sickle cell anemia.
- Although it is very rare to find sickle cell disease or trait in Caucasians, in patients undergoing myelosuppressive chemotherapies with growth factor support and with symptoms concerning for a possible sickle cell crisis, consideration should be made for testing for sickle cell trait or anemia.

## Figures and Tables

**Table 1:** Summary of other similar cases of sickle cell crises reported in patients receiving colony stimulating factors [17]^+^.

Study	Year	*N*	Sickle cell	Growth factor	Adverse event/complication	Outcome/comments
Pieters et al. [16]	1995	1	Sickle cell disease (SCD)	Intra-lesional G-CSF into a leg ulcer	Sickle cell pain crisis	Responded to supportive care

Abboud et al. [12]	1998	1	Sickle cell disease (SCD)	G-CSF*	Sickle cell pain crisis and possibly acute chest syndrome	Responded to supportive care and hydroxyurea

Adler et al. [14]	2001	1	Mild hemoglobin sickle cell	G-CSF	Sickle cell crisis, massive splenomegaly, and disseminated intravascular coagulation (DIC)	Death

Wei and Grigg [15]	2001	1	Compound heterozygous sickle cell/beta+ thalassemia	G-CSF	Sickle cell crisis leading to acute chest syndrome, DIC, and life threating multiorgan failure	Prolonged hospitalization for 8 weeks due to aforementioned complications

Kang et al. [11]	2002	8	8 SCT^x^ and 8 matched controls	G-CSF	No major adverse events reported; however, patients with SCT reported higher on the symptom score^#^	Responded to supportive care and analgesics

Richard et al. [22]	2005	3	Sickle cell disease (SCD)	G-CSF	2 patients developed sickle cell pain crisis	1 of the patients required parenteral narcotics

Kambel et al. [19]	2006	1	Hemoglobin SC (HbSC) disease	G-CSF	Severe sickle cell pain crisis requiring hospitalization	Split dose schedule for G-CSF and exchange transfusion to lower HbS levels from 20% to 6% were done to help complete treatment for patient's lymphoma

Rosenbaum et al. [18]	2008	5	4 with hemoglobin SS (HbSS) and 1 with hemoglobin SC (HbSC) disease	G-CSF	3 out of 5 patients develop symptoms suggestive of vasoocclusive crisis (VOC)	Responded to supportive care; only 1 patient required a packed red blood cell transfusion

Tormey et al. [21]	2008	1	Sickle cell disease (SCD)	G-CSF	Exchange transfusions done prior to prevent VOC	No significant complications reported

Onitilo et al. [20]	2003	1	Sickle cell disease (SCD)	G-CSF	No significant complications noted	Was hypertransfused to a hematocrit of more than 40% to prevent sickle cell crises

^+^Five of the studies presented here are already summarized in a table by Fitzhugh et al. Article as open access. Permission also obtained from the author [17].

*G-CSF: granulocyte colony stimulating factor (there are no reports with the pegylated form of the drug).

^x^SCT: sickle cell trait.

^#^Most common symptoms reported included nausea, headaches, myalgias, and fatigue. The average use of analgesics was also higher (13 tablets versus 6.25 tablets in patients with SCT versus controls resp.).

## Abbreviations

SCT: Sickle cell trait
G-CSF: Granulocyte colony-stimulating factor.
